# Optimizing a Solution Heat Treatment by Increasing the Cooling Rate of Directional Solidification for Ni-Based Superalloys

**DOI:** 10.3390/ma16093433

**Published:** 2023-04-28

**Authors:** Yanbin Zhang, Ling Qin, Bin Zhu, Haijun Jiang, Li Tan, Taiwen Huang, Bin Gan, Ziqi Jie, Lin Liu

**Affiliations:** 1 College of Materials Science and Engineering, Chongqing University of Technology, Chongqing 400054, China; tanli@cqut.edu.cn; 2 Chongqing Iron and Steel Research Institute Co., Ltd., Chongqing 400050, China; zhubincq@foxmail.com (B.Z.);; 3 Department of Petroleum Engineering, University of Wyoming, Laramie, WY 82071, USA; ling.qin4542002@outlook.com; 4 State Key Laboratory of Solidification Processing, Northwestern Polytechnical University, Xi’an 710072, China; 5 Beijing Key Laboratory of Advanced High Temperature Materials, Central Iron and Steel Research Institute, Beijing 100081, China; 6 School of Materials Science and Chemical Engineering, Xi’an Technological University, Xi’an 710021, China; jzq603@163.com

**Keywords:** Ni-based superalloy, solution heat treatment, solidification cooling rate, residual segregation

## Abstract

The solution heat treatment (SHT) of the third generation of single crystal (SC) Ni-based superalloys required up to 45 h and was expensive. In this study, SHT based on liquid metal cooling (LMC) was optimized to greatly reduce processing time. The experimental and simulation results showed that residual segregation was evidently reduced, e.g., from 2.12 to 1.22 for the most heavily segregated Re. This led to a 16.7% increase in creep life, more uniform microstructures, and a decrease in solidification and homogenization porosity by a factor of 3.4. Structural refinement, approximately 0.32 times, served as the underlying mechanism for this optimization, which reduced diffusion distance and increased homogenization efficiency during SHT.

## 1. Introduction

After the directional solidification of Ni-based SC superalloys, SHT was a vital step in adjusting the γ/γ′ microstructure and minimizing residual segregation. Refractory elements were added to deal with the problem caused by the rising operating temperature of turbine blades in aeroengines when SC superalloys were being developed. The addition of refractory elements such as Re, W, and Mo posed a challenge to the SHT due to their low diffusion coefficients [1]. To decrease the residual segregation and homogenize the microstructure, several studies [1,2,3,4] have raised the peak temperature of the SHT. However, defects such as pores, recrystallization, and eutectic occurred when the heat treatment temperature was higher than this incipient melting temperature, which adversely affected the high-temperature mechanical properties of the superalloys. In order to avoid incipient melting, the temperature was raised to its maximum. According to our previous work on a remelting SHT [5], although the temperature had climbed above the solidus temperature, the residual segregation remained severe.

So it was necessary to find an alternative optimization method. It was determined that the time required for homogenization during SHT was proportional to the square of the characteristic diffusion distance based on the sinusoidal distribution of elements between dendrites in the binary alloy model [6], indicating that the dendritic arm spacing (DAS) was an important factor affecting homogenization. In the pseudobinary system, this rule can theoretically be used to describe the elemental diffusion of SC superalloys. Liu et al. [7,8] found that the DAS and segregation improved when the cooling rate was high during directional solidification. Pistor et al. [9] stated that the technique of selective electron beam melting used to raise the cooling rate had a similar impact on the segregation. In theory, this provided a way to raise the homogenization efficiency in order to optimize SHT. The varied cooling rate was considered because it might lead to changes in the as-cast segregation of the elements as a result of solute redistribution. The back diffusion in the solid phase during solidification depends on the diffusion time and the diffusion distance. As the cooling rate raised, the solute diffusion time fell, which could increase segregation. Meanwhile, the dendritic structure was refined as well as the DAS and segregation was noticeably reduced, which is conducive to the back diffusion. In summary, the cooling rate could decrease residual segregation after SHT by improving both homogenization efficiency and as-cast segregation.

The freckle defect, which was thought to be caused by macro-segregation and thermal solute convection, was also frequently studied for its correlation to the cooling rate. It is revealed in refs [10,11] that the dendritic structure was refined, and freckle-type defects were avoided when the cooling rate of LMC directional solidification was high. As reported by Reinhart et al. [12], such a freckle drawback with a lower solidification cooling rate was caused by an increase in oscillation amplitude which was induced by the convective flow above the solidification front. Ren et al. [13] assumed that an increase in the cooling rate promoted the growth of the ternary dendritic arms, and it also removed the freckle defect by inhibiting solute transfer.

The mechanical properties, as an important characterization of the SHT effects, are also often studied in relation to the cooling rate. Liu et al. [14] illustrated that refined microstructure and a low fraction of the remaining eutectic, which were obtained by a high cooling rate, led to a significant improvement in the rupture life at 1000 °C and 235 MPa. This idea that a high cooling rate can have better solution effects is consistent with that in the literature [8]. However, it was demonstrated in refs [15,16,17] that although a high cooling rate benefited the as-cast state, this benefit almost disappeared after full SHT. Therefore, the effects of the cooling rate on the SHT during directional solidification were discussed. From that, it is concluded that the following optimization of SHT based on rapid directional solidification was limited.

In the current work, strategies like LMC solidification were successfully used to increase the cooling rate of directional solidification. The effects of the cooling rate on the SHT were studied. Furthermore, the SHT was successfully optimized in a short time. The microstructure, residual segregation, and creep life going through the LMC process and the optimized SHT were improved as compared to those after the High Rate Solidification (HRS) process and the standard SHT. These findings were expected to provide the basis for the potential applications of SC superalloys in the aviation and aerospace fields.

## 2. Materials and Methods

**Figure 1:**
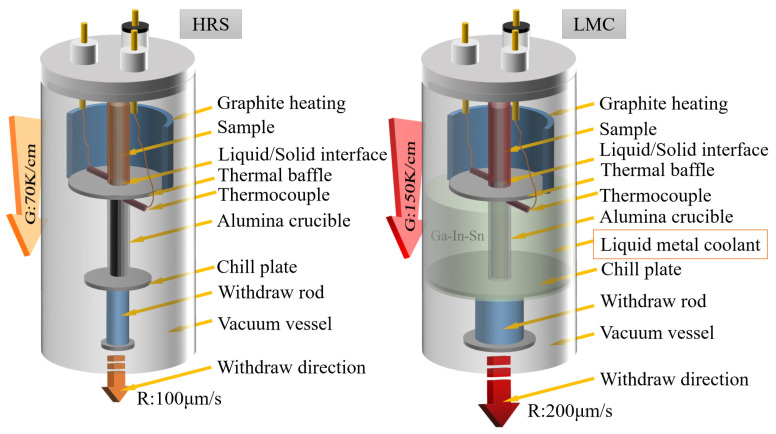
Schematic diagram of HRS and LMC directional solidification process. G is the temperature gradient around the liquid/solid interface; R is the withdrawal rate.

The nominal composition of the nickel-based SC superalloy in the present work is Ni-3.5Cr-9Co-1.6Mo-6W-5.7Al-8Ta-4Re-0.1Hf-0.001B-0.002C (wt. %). Two sets of experiments with different solidification cooling rates were designed, as shown in Figure 1. Two strategies were also used to change the solidification cooling rate, namely, different withdrawal rates and coolants with various thermal conductivity. In the control group, the HRS method was used to make samples at a withdrawal rate of 100 μm/s, and the temperature gradient was maintained at approximately 70 K/cm. The temperature gradient is measured using two W-Re5/26 thermocouples, and the thermocouples are protected by a metal-ceramic sleeve. In our experimental group, the temperature gradient of LMC samples at a withdrawal rate of 200 μm/s with a high-efficiency coolant Ga-In-Sn is stabilized at approximately 150 K/cm. The cooling rate is approximately equal to multiplying the temperature gradient by the withdrawal rate [8]. Therefore, the cooling rate of LMC samples was 3 K/s more than four times higher, as much as 0.7 K/s for HRS samples in the present study. The LMC samples led to a higher cooling rate during directional solidification.

All samples were etched with a solution of 20 mL HCl + 4 g CuSO_4_ + 20 mL H_2_O. Their microstructures were observed and analyzed using a Leica DM4000 optical microscope (OM) and a ZEISS SUPRA 55 scanning electron microscope (SEM). Ten pictures were randomly picked at 50 magnification for measuring solidification pores and heat treatment pores and 20 k magnification for measuring γ′ in each sample. The digital image analysis software SISC IAS V8.0 was used to calculate porosity area fractions and diameters of γ′ by distinguishing the contrast and brightness. The volume fractions of γ′ and pores were considered equal to their area fractions. In order to quantitatively investigate the uniformity of the size distribution of the γ′ particles, the coefficient of variation was used:

$$f_{cv}=\frac{\sqrt{\frac{1}{n-1}\sum_{i=1}^{n}{\left(d_{i}-d\right)}^{2}}}{\frac{1}{n}\sum_{i=1}^{n}d_{i}}$$

In this equation, di is the diameter of γ′, and d is the mean diameter of γ′. Thermo-Calc & DICTRA software with the MOBNI3 and TCNI7 database was used to simulate the phase transition temperature and the diffusion processes during SHT. The diffusion distance was considered as half of the average primary DAS, which was obtained by the area counting method. A point matrix scanning technique was used to obtain the initial chemistry profiles by an Oxford X-max20/Inca 250 energy dispersive spectroscopy (EDS). In this technique, 100 points were measured to characterize the whole dendritic structure. An area of 300 μm × 300 μm with a spacing of 30 μm for the HRS samples and an area of 100 × 100 μm^2^ with a spacing of 10 μm for the LMC samples were scanned in a dwelling time of 120 s. The SEM accelerating voltage was 20 kV. In order to avoid overlapping lines, a characteristic X-ray K line was used for Al and L lines were used for Ta, W, and Re. The component of the dendritic core over the interdendritic area was used as the segregation ratio. Since incipient melting occurs in the interdendritic areas (IAs), the composition of the IA determines the incipient melting temperature. The compositions obtained by the EDS in the IA after the HRS and the LMC process were used to calculate the incipient melting temperature by Thermo-Calc software.

Prior to heat treatment, all samples were placed in quartz glass tubes with a 10^−4^ Pa vacuum, then filled with argon gas at atmospheric pressure and subsequently sealed for protection. The standard SHT-HT1 was 1250 °C/1 h + 1290 °C/1 h + 1300 °C/2 h + 1310 °C/3 h + 1320 °C/5 h + 1330 °C/15 h + air cooling. The standard aging treatment was 1180 °C/4 h + 870 °C/24 h + air cooling. All heating rates were 10 °C/min. SHT and aging experiments were performed in an industrial furnace with a temperature variation of ±5 °C. After SHTs and the aging treatment, samples subjected to the creep rupture test were machined with a diameter of 6 mm and a gauge length of 30 mm. The rupture tests were conducted at 1100 °C and 150 MPa. Two identical specimens were tested for each case.

## 3. Results and Discussion

**Figure 2:**
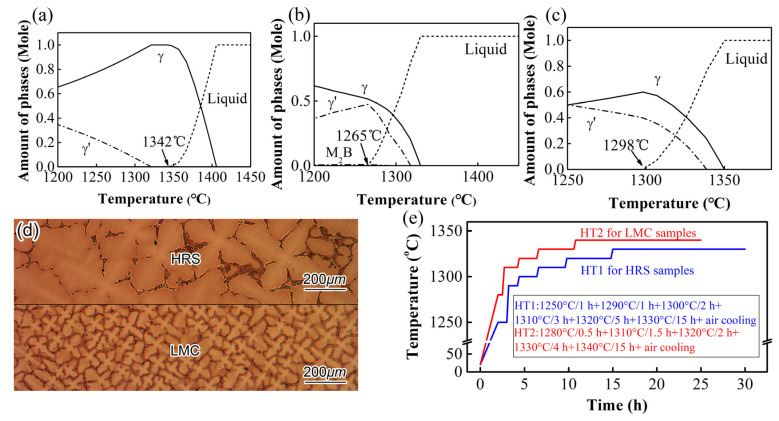
(**a**–**c**) simulation results of phase transition temperature; (**d**) OM micrograph of as-cast dendritic structures; (**e**) schematic drawing of SHTs.

Experimental methods such as Differential Scanning Calorimetry (DSC) were usually used to obtain the incipient melting temperature. However, it was indicated in previous work [18] that the DSC could hardly measure the incipient melting of boride for boron-bearing superalloys due to very little enthalpy change. Thus, simulation was used to analyze the different incipient melting temperatures for the alloys with different segregation, i.e., high segregation for HRS, low segregation for LMC and no segregation for average composition. Details of the simulation can be seen in the Materials and Methods part. Figure 2a shows the phase transition temperature of the average composition of the master alloy representing full homogenization. Its solidus temperature is 1342 °C, which is the limit of the peak temperature during all SHTs. Figure 2b,c show that the incipient melting temperatures are 1265 °C after the HRS process and 1298 °C after the LMC process. The high incipient melting temperature was caused by the less segregation after the LMC process. Thus, the initial SHT temperature of the LMC samples could be higher than the HRS samples.

Figure 2d shows the dendritic structures after different directional solidification processes. The primary DAS of LMC samples is 83 μm, which is finer than that of HRS samples (260 μm) due to the higher cooling rate in the LMC process. As a result, the diffusion distance after the LMC process is shorter, and the homogenization process takes less time. Following the steps of the SHTs are designed and shown in Figure 2e. According to the analysis above, the standard SHT-HT1 was optimized to HT2 for LMC samples with higher temperatures (1280–1340 °C vs. 1250–1330 °C) and a shorter period (25 h vs. 30 h).

**Figure 3:**
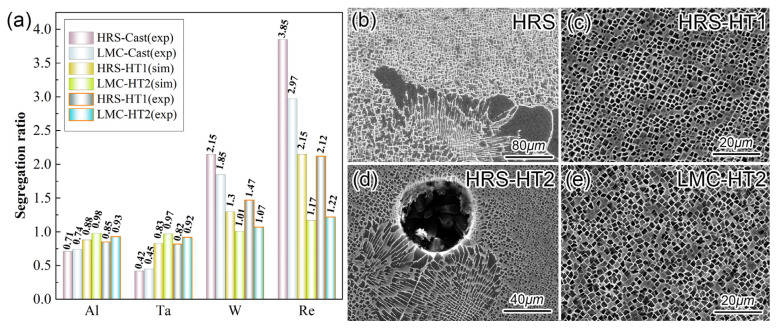
(**a**) simulation (sim) and experiment (exp) results of segregation ratios; (**b**–**e**) SEM micrograph of microstructures after different solidification and SHT processes.

Figure 3a shows the segregation ratios after different solidification and SHT processes. The Re tends to segregate most severely. The as-cast segregation after the LMC process is lower than that after the HRS process, which is consistent with prior studies [14,15]. The experiment results are similar to the simulation results. Despite the shorter homogenization time of HT2, the residual segregation after the LMC-HT2 is lower than that after the HRS-HT1. The reasons are the higher SHT temperature and the shorter diffusion distance, which will be discussed later. Inasmuch as the dissolution of the eutectic was ignored in the simulation, the homogenization process was accelerated, and the residual segregation was a little bit less severe than the experimental results.

Figure 3b shows the as-cast microstructure with a huge eutectic. Figure 3c–e shows the microstructures after HRS-HT1, HRS-HT2 and LMC-HT2. Comparing Figure 2b,c,e, the primary γ′ and γ/γ′ eutectic after the HRS process were totally dissolved by HT1 and HT2. In Figure 3d, the initial SHT temperature of HT2 1280 °C exceeds the incipient melting temperature of the HRS sample 1265 °C and incipient melting occurs. While the initial SHT temperature of HT2 is 1280 °C below the incipient melting temperature of the LMC sample, 1298 °C, and, thus, the microstructure after LMC-HT2 in Figure 3e is similar to that in Figure 3c without incipient melting. These experimental results are consistent with the simulation results in Figure 1, that the incipient melting temperature of LMC samples is higher than that of HRS samples. HT2 is, thus, suitable for the LMC samples. Note that the peak temperature in the HT2 is 1340 °C, which almost reaches the limit of 1342 °C shown in Figure 2a, and the incipient melting is still avoided.

**Figure 4:**
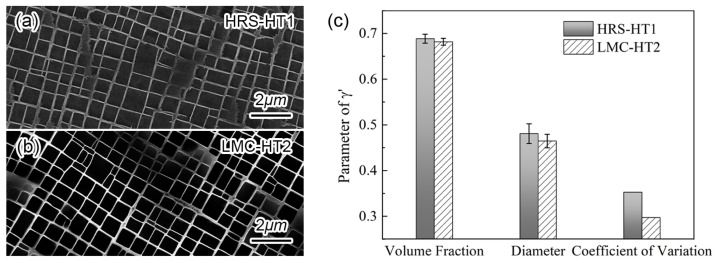
Comparison of (**a**,**b**) SEM micrograph of microstructures, (**c**) parameters of γ′ between HRS-HT1 and LMC-HT2 samples.

Figure 4a,b show the γ′/γ′ structures after the SHTs and aging treatments. After the aging treatments, the shape of γ′ in two samples became cubic. Compared with γ′ after HRS-HT1, the distribution of γ′ after LMC-HT2 is more uniform. Figure 4c shows the parameters of γ′, including the volume fraction, diameter and coefficient of variation. From HRS-HT1 to LMC-HT2, with the reduction of residual segregation, the volume fraction and average size of γ′ are hardly changed, and the coefficient of variation of γ′ is reduced, which means the size distribution of the γ′ particles is more homogeneous.

**Figure 5:**
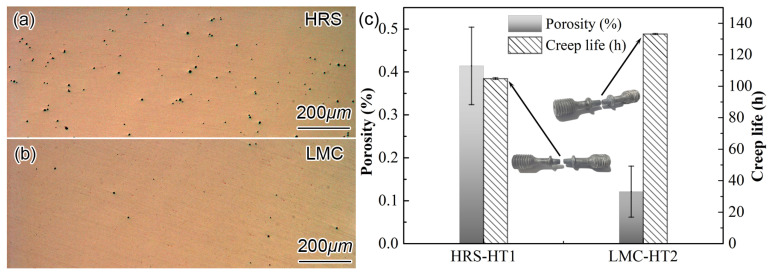
Comparison of (**a**,**b**) OM micrograph of micro-pores after heat treatments, (**c**) porosity and creep life at 1100 °C/150 MPa between HRS-HT1 and LMC-HT2 samples.

Figure 5a,b display the micro-pores after the SHTs and aging treatments. Their porosity statistics are shown in Figure 5c. It is indicated that the porosity in the LMC-HT2 sample is significantly less than that in the HRS-HT1 samples. The porosity consists of two parts, the solidification porosity and the homogenization porosity. The solidification porosity after the LMC process is less than that after the HRS process due to the high cooling rate. The homogenization rate of LMC-HT2 is much faster (see Figure 3a); thus, the homogenization porosity caused by the imbalance of the diffusion flux is much less than that after the HRS-HT1. Less porosity is beneficial to mechanical properties. Figure 5c also shows the creep life for each rupture test at 1100 °C and 150 MPa. Samples with the same condition were tested twice, and their difference is very small, as can be seen from the error bars. In Figure 5c, the creep life of the LMC-HT2 sample is longer than that of the HRS-HT1 sample. This indicates that the decrease of the residual segregation and porosity strengthens the creep property after LMC-HT2. This is consistent with former refs [19,20,21]. Compared to the former research, however, the improvement of creep life is less than expected. This is probably due to the volume fraction, and the diameter of the γ′ changed little, as shown in Figure 4c.

The residual segregation directly affects the mechanical properties of the alloy by changing the shape, size and content of the γ′ phase. As shown in Figure 4c, reducing the residual segregation resulted in a more uniform size distribution of γ′ in the dendrite and interdendritic. The uniformly distributed γ′ would form a continuous γ′ rafting structure during creep, which effectively hinders the climbing of dislocations in its vicinity, while the homogenization of the composition would produce a uniform and dense dislocation network, which hinders the dislocations motion and effectively improves the high-temperature creep properties of the alloy. In addition, residual segregation affects the mechanical properties of the alloy by altering the γ′ rafting process. Huang et al. [22] indicated that the creep life of a fourth-generation Ni-based single crystal superalloy at 1140 °C/137 MPa was prolonged due to the slower rafting process. It is illustrated that the slower rafting process was caused by the slightly coarsened γ′ phase and homogenization of refractor elements.

It is well known that SHT has three roles: (1) dissolving primary γ′ particles, (2) dissolving γ/γ′ eutectics, (3) depressing residual segregation. Eliminating the primary γ′ and γ/γ′ eutectics is an important but relatively simple task for SHT. As shown in Figure 3c primary γ′ particle and the γ/γ′ eutectics are totally dissolved by HT1, but severe residual segregation still remains in Figure 3a.

The size and volume fraction of γ′, though significant for the mechanical property of alloy, could be adjusted by means of the aging treatment, while the microstructure uniformity of γ′ can only be tuned by reducing the residual segregation through SHT. Hence, the significant role of the SHT is to reduce the residual segregation for high-generation superalloys with large amounts of refractory elements, especially Re.

In the present work, for SHT, the standard SHT-HT1 and optimized SHT-HT2 are used; but for aging treatment, only standard aging treatment is used. Since the standard aging treatment designed for HRS-HT1 was used for LMC-HT2, the volume fraction and diameter of the γ′ changed little, which means the utilization of the optimized SHT was insufficient. Our further work is to optimize aging treatment for LMC alloy to pursue a greater increase in creep strength. Nonetheless, the creep life of the alloy has been improved to some extent due to the less residual segregation, even though the aging treatment is not optimized yet.

**Figure 6:**
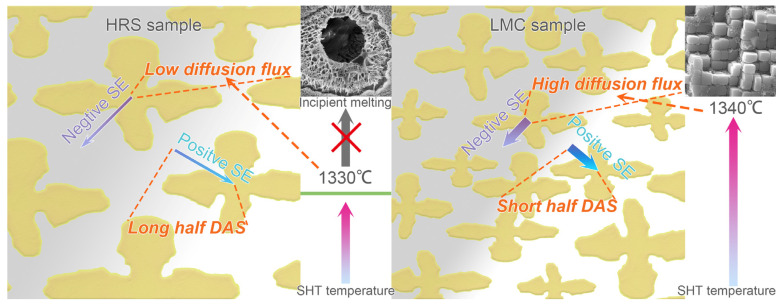
The schematic illustration of different homogenization rates for segregation elements (SE) in HRS and LMC samples.

The results in Figure 3a shows that the homogenization rate during LMC-HT2 is considerably faster than that during HRS-HT1. Two reasons are illustrated in Figure 6: (1) higher SHT temperature to boost the diffusion of SE due to the wider limit for LMC sample to avoid incipient melting; (2) shorter diffusion distance, which makes the homogenization process less time-consuming. The segregation ratios after LMC-HT2 were lower than those after the two remelted SHTs in the previous investigations, for example, from 1.35 [2] and 2.01 [5] to 1.22 for Re, and the peak temperatures of the latter two even reached 1360 °C which is noticeably higher than the incipient melting temperature. This comparison indicates that for optimizing SHT, increasing the cooling rate to shorten the diffusion distance and increase the temperature is more effective than increasing the temperature alone. Thus, it demonstrated that a rapid cooling rate has remarkable effects on the homogenization rate during SHT.

However, up to date, in most cases, the SHT of each alloy does not depend on its specific solidification parameters, such as solidification cooling rate. This would lead to two absurd situations. For starters, substantial time would be wasted in the low SHT temperature for alloys with shorter DAS. This is most probably what happened in the refs [16,17] since all samples with varying DASs were fully homogenized after the same SHT. Secondly, for alloys with longer DAS, incipient melting occurs, as shown in Figure 3d. Therefore, SHT should be optimized for superalloy with different solidification cooling rates.

The effects of parameters of directional solidification and SHT on solution effects have usually been investigated separately. Although a few studies [7,10,12,18] have attempted to examine the connection between solidification and SHT in terms of solution effects, it is debatable whether the directional solidification process has an impact on solution effects because controlled experimental studies on both sides have used the inappropriate identical SHT in varying the cooling rate of solidification. For the studies [7,18] that believed no impact, the use of the identical SHT caused two problems: (1) too small an effect to be discovered without the optimization like increasing solution temperature, (2) although the fast cooling experimental group did not need such long time SHT, same results were obtained due to the SHT time long enough for the homogenization of slow cooling group. Though Yan and Elliott et al. [10,12] argue solidification cooling rate affected SHT results, a considerable amount of time was wasted without optimization, as discussed in the preceding paragraph.

This paper utilizes the effects of solidification cooling rate on SHT, such as the change of incipient melting temperature and the variation of diffusion distance during homogenization. As the cooling rate is raised, the matching SHT optimization approach is proposed. It should be noted that these effects and similar optimization approaches could be utilized in other as-cast alloys when their microstructure was refined, and the segregation was limited, allowing for higher phase transition and heat treatment temperatures.

## 4. Conclusions

In this paper, two strategies, including a fast withdrawal rate and a high-efficiency coolant, are used to increase the cooling rate during directional solidification. SC superalloys with very fine dendritic structures are successfully obtained. The effects of the solidification cooling rate on SHT are exploited. Accordingly, the time and temperature were adjusted to optimize the SHT process to improve the solution effects and high-temperature creep properties. These findings are expected to propose an optimization method of SHT for the potential applications of advanced nickel-based SC superalloys for aero-engines. The important and quantitative findings are as follows:

1. A solidification cooling rate of more than four times higher than LMC samples was caused by LMC directional solidification with a high withdrawal rate compared with HRS samples. Thus, it is found that the differences between HRS samples and LMC samples are the primary DAS (260 μm vs. 83 μm) and the incipient melting temperature (1265 °C vs. 1298 °C). Accordingly, HT1 for HRS samples was optimized to HT2 for LMC samples with higher temperatures (1280–1340 °C vs. 1250–1330 °C) and shorter duration (25 h vs. 30 h).
2. The effects of the solidification cooling rate on SHT are exploited. Compared to the HRS-HT1 samples, residual segregation of LMC-HT2 samples is significantly minimized; for example, the segregation ratio of Re is reduced from 2.12 to 1.22. Less residual segregation results in a more uniform microstructure and a porosity reduced by a factor of 3.4, both of which are beneficial to the creep property.
3. The dependence of the solidification cooling rate on the creep lifetime was investigated. The creep life of LMC-HT2 samples at 1100 °C and 150 MPa is increased from 104.9 h for HRS-HT1 samples to 133.2 h.

## Data Availability

Not applicable.
